# Socioeconomic factors analysis for COVID-19 US reopening sentiment with Twitter and census data

**DOI:** 10.1016/j.heliyon.2021.e06200

**Published:** 2021-02-06

**Authors:** Md. Mokhlesur Rahman, G.G.Md. Nawaz Ali, Xue Jun Li, Jim Samuel, Kamal Chandra Paul, Peter H.J. Chong, Michael Yakubov

**Affiliations:** a University of North Carolina at Charlotte, NC 28223, USA; b University of Charleston, WV 25304, USA; c Auckland University of Technology, Auckland 1010, New Zealand; d Wilmington University, DE 19720, USA; e Khulna University of Engineering & Technology (KUET), Khulna 9203, Bangladesh

**Keywords:** COVID-19, Coronavirus, Reopen, Sentiment analysis, Twitter, Census, Binary logit model

## Abstract

Investigating and classifying sentiments of social media users (e.g., positive, negative) towards an item, situation, and system are very popular among researchers. However, they rarely discuss the underlying socioeconomic factor associations for such sentiments. This study attempts to explore the factors associated with positive and negative sentiments of the people about reopening the economy, in the United States (US) amidst the COVID-19 global crisis. It takes into consideration the situational uncertainties (i.e., changes in work and travel patterns due to lockdown policies), economic downturn and associated trauma, and emotional factors such as depression. To understand the sentiment of the people about the reopening economy, Twitter data was collected, representing the 50 States of the US and Washington D.C, the capital city of the US. State-wide socioeconomic characteristics of the people (e.g., education, income, family size, and employment status), built environment data (e.g., population density), and the number of COVID-19 related cases were collected and integrated with Twitter data to perform the analysis. A binary logit model was used to identify the factors that influence people toward a positive or negative sentiment. The results from the logit model demonstrate that family households, people with low education levels, people in the labor force, low-income people, and people with higher house rent are more interested in reopening the economy. In contrast, households with a high number of family members and high income are less interested in reopening the economy. The accuracy of the model is reasonable (i.e., the model can correctly classify 56.18% of the sentiments). The Pearson chi-squared test indicates that this model has high goodness-of-fit. This study provides clear insights for public and corporate policymakers on potential areas to allocate resources, and directional guidance on potential policy options they can undertake to improve socioeconomic conditions, to mitigate the impact of pandemic in the current situation, and in the future as well.

## 1 Introduction

**Figure 1:**
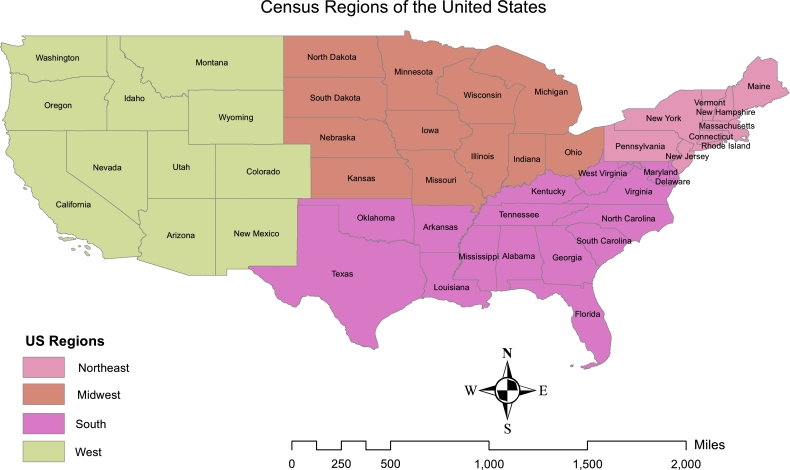
Census regions of the US.

There is a critical need to understand public sentiment concerning post-COVID-19 economic reopening and the associated socioeconomic factors. First documented in the mid-1960s, there have been seven identified Coronaviruses in the world that can infect humans. Within the human population, Sudden Acute Respiratory Syndrome – Coronavirus-2 (SARS-Cov-2) which causes the disease known as COVID-19 is the fifth endemic Coronavirus including 229E, HKU1, NL63, and OC43 [1], [2]. COVID-19 is the highly infectious disease caused by the third identifiable Coronavirus that emerged among humans in the last two decades. Among the three most recent Coronaviruses, the Severe Acute Respiratory Syndrome Coronavirus (SARS-CoV) emerged in China between November 2002 and July 2003 spreading to 17 countries with a fatality rate of 9.6% [3], [4]. In 2012, Middle East Respiratory Syndrome Coronavirus (MERS-CoV) was discovered in the Middle East affected 24 countries with a fatality rate of 34.4% [5], [4]. COVID-19 was first identified in Wuhan, China, and by the end of December 2019, had already affected over 10 million people in 213 countries with a fatality rate, that had reportedly almost reached 10% among the closed cases [6]. COVID-19 is a highly infections and deadly disease with widespread transmission and significant impacts to the human physical, emotional, and mental health. COVID-19 has also had an impact on the economy, and the way society acts and responds on a daily basis [7], [8]. To control the spread of COVID-19, the federal government declared statewide emergency and state governments implemented a stay-at-home-order, imposed restriction on mass gathering and non-essential movements. Consequently, people are confined at home with constant fear and uncertainty. These mitigation measures adopted by the communities are rarely practiced and have limited setting, causing serious upheaval of attitudes, believes and willingness of the people [9]. Additionally, the unemployment rate showed an increasing trend. To tackle economic depression, many people are arguing to reopen the economy. This study investigates the sentiment of people towards reopening the US economy and finds the underlying socioeconomic factors that are associated with prominent public sentiment. We combine the US public Twitter sentiment and the US demographic information from the US Census Bureau. The studied US Census regions are shown in Fig. 1.

Continuous lockdown is not a long-term solution for any country. Deliveries of necessary medical supplies including Personal Protection Equipment (PPE) and lab equipment are hindered because of the travel restrictions. The supply of necessary foods and household necessities has been halted due to the affected global supply chain system. According to the US Department of Labor, the US economy has lost about 20.5 million jobs with a surge of the unemployment rate to 14.7% in April 2020 which surpassed the post-World War II record of 10.8% in 1982 [10]. Labor Body of the United Nations (UN) reported that the world is expected to witness a 6.7% elimination of working hours, which is equivalent to 195 million jobs, in the second quarter of 2020 due to economic disruption triggered by the COVID-19 pandemic [11]. This economic downfall describes the depth of the economic recession worldwide caused by COVID-19 and related lockdown measures (e.g., stay-at-home order, restriction on public gathering, workplace and school closing) and travel restrictions. Moreover, the persistence of the Coronavirus outbreak is posing a threat to the education systems. Schools, colleges, and universities are closed because of the virus which is hampering in-seat education and hands-on laboratory work severely [12]. Many local, as well as international students, are the worst victims because they are considered as the potential risk of COVID-19 importation [13], [14]. However, taking into account the adverse effects of COVID-19, people are craving to go back to normal life and regular activities. The emotionally challenging but true reality is that, amidst the fear of pandemic, people need to go out, do their jobs and run the economy.

Past research investigated fear and trust sentiments of the people towards reopening the economy in the US using exploratory textual analytics, textual data visualization, and hypothesis testing techniques [8]. Similarly, past research has also investigated and classified sentiments of the users (e.g., positive, negative) towards an item, situation, and system [7]. However, they rarely discussed the underlying socioeconomic factor associations for such sentiments. This study attempts to explore the socioeconomic factor associations for positive and negative sentiments of the people about reopening the economy in the US in the middle of COVID-19 global crisis considering the situational uncertainties (i.e., changes in work and travel pattern due to lockdown policies) and depressions of the people. To control the massive spread of COVID-19 cases by forcing people to stay isolated, the federal and state governments have imposed ‘stay at home’ order and ‘state emergency’. Consequently, dramatic changes have been observed in the daily lifestyle and travel patterns of the people.

People are adversely affected by the many COVID-19 related anti-transmission measures taken by the state and federal governments. Numerous companies and business are already closed permanently. Many people have lost their jobs and undergoing uncertainty and depression. To address this problem state governments have already started to reopen the economy with different stages of operation (i.e., the complete business operation to the limited scope of operation). However, there is a counter-argument to delay reopening the economy since it will allow people to interact with each other and make different types of trips and consequently the states will face serious difficulties to manage the situations. Many persons are expressing their opinions and aspirations on social media for and against the new normal reopening. This study aimed to understand the sentiment of the people towards the reopening the economy and investigate the reasons that influence their positive and negative sentiments about the imminent new normal reopening amid COVID-19 pandemic situations. Our contributions through this paper are as follows:

• Novel data assimilation: We have collected Twitter data to analyze the public sentiment about reopening the US economy amidst COVID-19 pandemic and integrated those Twitter-generated sentiment results with the US Census data [15] to understand what socioeconomic factors influence individuals expressing positive or negative sentiments about reopening.
• Early application of methodology: We have provided a detailed methodology of this study from data collection to results reporting and discussion with a visual representation. Researchers who want to conduct similar studies by collecting data from social media (e.g., Twitter, Facebook, LinkedIn, Instagram, and news agency) about a real-world social event (e.g., man-made and natural disasters, political affairs, religious and racial conflicts) and integrating social media data with Census or household based survey to get some insights, can adopt this methodology.
• Parsimonious logit model: We have modeled a binary logit model with Twitter sentiments and Census data to better understand the most influential features in reopening public sentiment from a set of total 47 initial features. The developed model has over 56% accuracy to identify the sentiments with a high goodness-of-fit.
• Timely recommendations: Based on our research findings, we have made some suggestions/recommendations for policymakers where to allocate resources to improve the socioeconomic situations of the country and reduce the post COVID-19 sufferings of people.

The rest of the paper is organized as follows: Section 2 discusses the literature review of this study. Section 3 demonstrates data handling process and modeling binary logit method. Section 4 discusses about the results and findings of this study. Finally, we conclude this paper in Section 5.

## 2 Literature review

The present research seeks to understand factors that support reopening, expressed through positive sentiment towards reopening. The goal of the present study is to extend past research which has demonstrated the prominence of positive sentiment towards reopening, though there exists a fair amount of negative sentiment as well [8]. Though Twitter data has been intrinsically analyzed extensively and also contextualized to numerous domains, yet past research has not combined recent Twitter data with demographic data to model potential relationships between sentiment classes based on tweets and COVID-19 relevant data [16], [17], [18]. To provide a meaningful research basis for such an exercise, the present study conducted focused literature review on relevant topics, as summarized in the following sections on Twitter analytics, human behavior and sentiment analysis.

### 2.1 Twitter data analytics

Twitter data has been used for a wide range of analyses, including but not limited to healthcare, retail marketing, stock trading, education and politics [19], [20], [21], [22], [18], [23], [24], [25], [26]. Twitter data offers a wide range of variables depending on the download programming interface or mechanism used. The use of rSentiment package in R allows for the download of 90 variables(including variables such as type of device used, stated location, hast tags, display text width, reply to user ID, quote, retweet, favorite count, retweet count, URLs used, followers count, and date and time, to name a few) providing a rich array of variables associated with each post, which can be used to better understand the sentiment associated with the tweet [27]. In addition to the rich diversity of Twitter variables that lend themselves to analysis, Twitter posts or “Tweets” contain textual which are not easily manipulated, and therefore require specialized analysis. Additional elaboration on the specific steps used is provided under the methods section (Section 3.2).

### 2.2 Human behavior and sentiment

The present study utilizes public sentiment derived from social media posts as a key variable, and this is supported by extant research which has used sentiment analysis for diverse research purposes such as decision support, education, politics, opinion mining, data visualization, healthcare and hate crimes, and the importance of education, gender sensitivity and motivation [7], [23], [25], [28], [29], [30], [31], [32], [33], [34]. These studies have used a wide range of methods, tools and languages such as Python and R, and their associated libraries, to estimate sentiment from social media posts. Sentiment estimation can be broadly classified into two buckets, the first is the assignment of a score which ends to be continuous within a given range of an approximate minimum negative value (such as -2) to an approximate maximum positive value (such as +2), and the second consists of binary classification mechanisms (usually into positive and negative sentiment classes) or categorical classifications of data into sentiment classes such as fear, trust and sadness. For the purposes of this study, we use the R statistical modeling language from CRAN, and its associated sentiment analysis packages called sentimentr and syuzhet [35], [36], [37]. Past studies have also developed customized mechanisms to study human characteristic traits such as dominance, with the potential for corresponding emotions of anger and elation expressed through textual communications, and identified via manual or automated textual analytics [38], [39].

### 2.3 US census data and socioeconomic analysis

To find out the socioeconomic factor associations of reopening sentiments, this study also collected socioeconomic and demographic (e.g., income, education, age, family type and size, race, housing type etc.) information of the people from the American Community Survey (ACS) [15]. Moreover, data on the factor of the built environment (e.g., population density) was collected from ACS to assess the impacts of urban form on the Coronavirus fears and reopening sentiments of the people. ACS is conducted by the US Census Bureau each year to collect vital information about the citizens. The data is free, publicly available, and considered as an important source of information for researchers from different disciplines. Many previous studies collected information from ACS and leveraged with Twitter data to analyze sentiment of the people in the arena of public health [30], urban spaces [40], politics [41], [42], disasters management [43], racial conflicts [32], [44], and gender disparity [45]. Thus, linking Twitter data with Coronavirus data is a common practice among the researchers to evaluate the impacts of socioeconomic and demographic characteristics on the sentiments of the people towards a subject of interest. Moreover, the name of the four regions from where the tweets were generated was collected based on the US Census to evaluate regional impacts on reopening sentiments [46]. This study also collected information on the number of cases and deaths from Worldometer [47] to understand how the severity of Coronavirus influence the sentiment of the people about reopening the economy. Considering the unavailability of the exact location of Twitter users, state averaged Census and Coronavirus data were collected and integrated with Twitter data to perform the analysis.

### 2.4 Reopening risks

Restarting the US economy could cause a rise in Coronavirus infections and the COVID-19 related mortality rate [48], and therefore it is necessary to manage reopening risks with due consideration for socioeconomic factors. A study using an agent-based Susceptible, Exposed, Infected, and Recovered (SEIR) model evaluated the impacts of alternative lockdown measures and reopening scenarios on coronavirus cases and deaths in Florida, Georgia, and Mississippi [49]. The model assessed that imposing lockdown one week earlier in all the three states could have saved hundreds of lives from COVID-19. However, to reopen the economy even with a limited capacity, it is required to reduce the population contact down to 20-25% and implement strict social distancing measures along with the use of personal protective equipment [49]. Besides, a robust testing capacity would help the policymakers to estimate more precise reopening dates and it would be beneficial to detect and isolate asymptomatic carriers in a quicker manner after reopening. Various empirical evidence, trials, and observations suggested that the proper use of medical masks, combined with other non-pharmaceutical interventions (NPIs) such as thorough handwashing and strict social distancing, testing, contact tracing, and quarantine, is effective to reduce the transmission of COVID-19. Therefore, the deployment of masks in the public zones combined with other measures may eventually help in reopening the economy and transitioning into the post COVID-19 world [50], [51]. Though many such risks are systemic, any effective risk mitigation strategy could be enhanced with the identification and use of most relevant socioeconomic factors.

## 3 Data and study methods

### 3.1 Data

**Figure 2:**
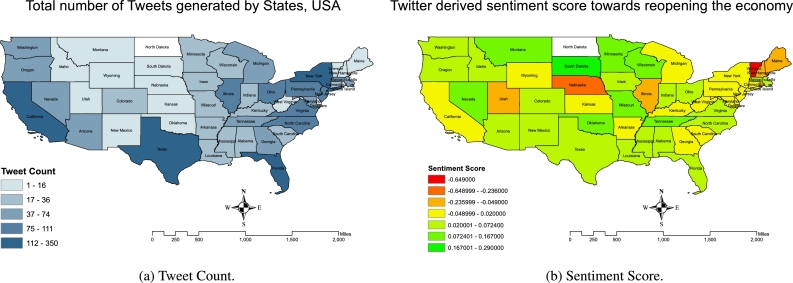
Number of tweets and Twitter-driven sentiment score.

This study uses Twitter data collected between April 30, 2020, and May 08, 2020, to understand the sentiment of the people towards the reopening of the US economy [8]. A total of 293,597 tweets with 90 variables were downloaded using the keyword “reopen”. The detailed methodology of data acquisition, saving, cleaning, and filtering for obtaining a subset of them that were generated from different states of the US has been described in Section 3.2. A major reduction in the quantity of usable Tweets was based on two critical cleaning processes and filters for removing spam Tweets with URLs, and Tweets without clear geographical tags for the US. After systematic cleaning and filtering, a final dataset consisting of 2507 tweets and twenty-nine variables were used for sentiment analysis and exploring the socioeconomic factor associations that influence sentiments of the people towards reopening the economy. Fig. 2a shows total number of tweets collected from each state in the US. The figure demonstrates that a large number of tweets were generated from the Western and Northeastern regions of the US. Most of the tweets were collected from California, Texas, New work, Florida, Pennsylvania, Illinois, Ohio, North Carolina, Virginia, New Jersey, Arizona, and Nevada. In contrast, no tweets were collected from North Dakota. Fig. 1 displays the Census regions of the US with the states in each region. To investigate regional influence on reopening sentiment, regional dummies (0 or 1) were created and included in the model.

Sentiment analysis is a popular topic of research among the researchers after collecting data from Twitter, webpage, product reviews, newspaper etc. [28], [52], [53], [54]. The primary objective of the sentiment analysis is to investigate opinions, attitudes, and emotions of the users (i.e., positive or negative feeling) towards a subject matter of interest (e.g., entity, person, issue, event, and topic). Sentiment analysis is beneficial for both customers and service providers to improve the mutual relationships [28]. In this study, Twitter data was analyzed to understand the sentiments of the Americans towards the new normal reopening amidst COVID-19 outbreak. Using R packages Syuzhet and sentimentr, sentiments were classified and assigned a score based on matching keywords, word sequences, and prewritten lexicons. Sentiment score was assigned within a range of -2 to +2. The maximum negative value indicates negative sentiment, whereas the maximum positive value indicates positive sentiment, with a score 0 indicates neutral sentiment. Results from a preliminary analysis showed that about 48.27% of the users expressed positive sentiment. In contrast, about 36.82% and 14.92% of the users expressed negative and neutral sentiments, respectively. Fig. 2b shows spatial distribution of the Twitter-driven sentiment score towards reopening the economy. It indicates that most of the states showed positive sentiment towards reopening the economy. Calculating an average score value for the US, we found that mean value of the sentiment score is 0.0271 considering positive, negative and neutral tweets. Thus, most of the Twitter users posted positive information about the reopening. States with highest positive sentiments include South Dakota, Wisconsin, Oklahoma, Montana, Tennessee, Minnesota, and Missouri. On the other hand, Vermont, Nebraska, Utah, Maine, Illinois, New Hampshire, and Rhode Island showed the highest negative sentiment towards the reopening. Table 1 represents the descriptive statistics of the variables used in the binary model.

**Table 1:** Descriptive statistics of the variables.

Variable	Variable description	Measure	Mean	SD	Min	Max
Tweet characteristics (2501 Tweets)						
Sentiment	Sentiment type	Dummy (0, 1)	0.48	0.50	0.00	1.00
TW	Number of words in the tweet	#	169.35	81.49	6.00	296.00
Regional Dummies (4 regions)						
NE	Northeast regions	Dummy (0, 1)	0.20	0.40	0.00	1.00
MW	Midwest region	Dummy (0, 1)	0.17	0.37	0.00	1.00
WEST	West region	Dummy (0, 1)	0.26	0.44	0.00	1.00
State-level socioeconomic characteristics and population density (50 States of the US and Washington D.C, the capital city of the US)						
L_FHH	Log of Percentage of family household	%	4.18	0.05	3.77	4.32
AFS	Average family size	#	3.26	0.17	2.85	3.62
EDU2	Percentage of persons with high school graduate and some college	%	47.10	4.73	30.02	59.14
EDU3	Percentage of persons with an associate degree	%	8.25	1.19	3.01	11.48
AGE2	Percentage of person Age under 18	%	22.25	1.66	18.10	29.50
WP	Percentage of white persons	%	75.48	8.39	25.60	94.60
OCH	Percentage of owner-occupied housing	%	62.66	5.77	41.80	72.90
PWHI	Percentage of persons under age 65 years without health insurance	%	10.22	4.35	3.20	20.00
LF	Percentage of the population age 16 years+ in the labor force	%	62.94	2.73	53.10	69.70
L_POPDEN	Log of Population density	Persons/mile2	5.24	0.99	0.18	9.20
CASES	Total number of cases per 1 million population	cases/1M people	5856.56	5466.00	458.00	19479.00
PR	Persons in poverty (Poverty rate)	%	13.07	2.06	7.60	19.70
MHHI	Median household income (2014-2018)	$	61962.71	10709.20	48.49	82604.00
GR	Median gross rent (2014-2018)	$	1084.67	217.14	711.00	1566.00

### 3.2 Study methods

**Figure 3:**
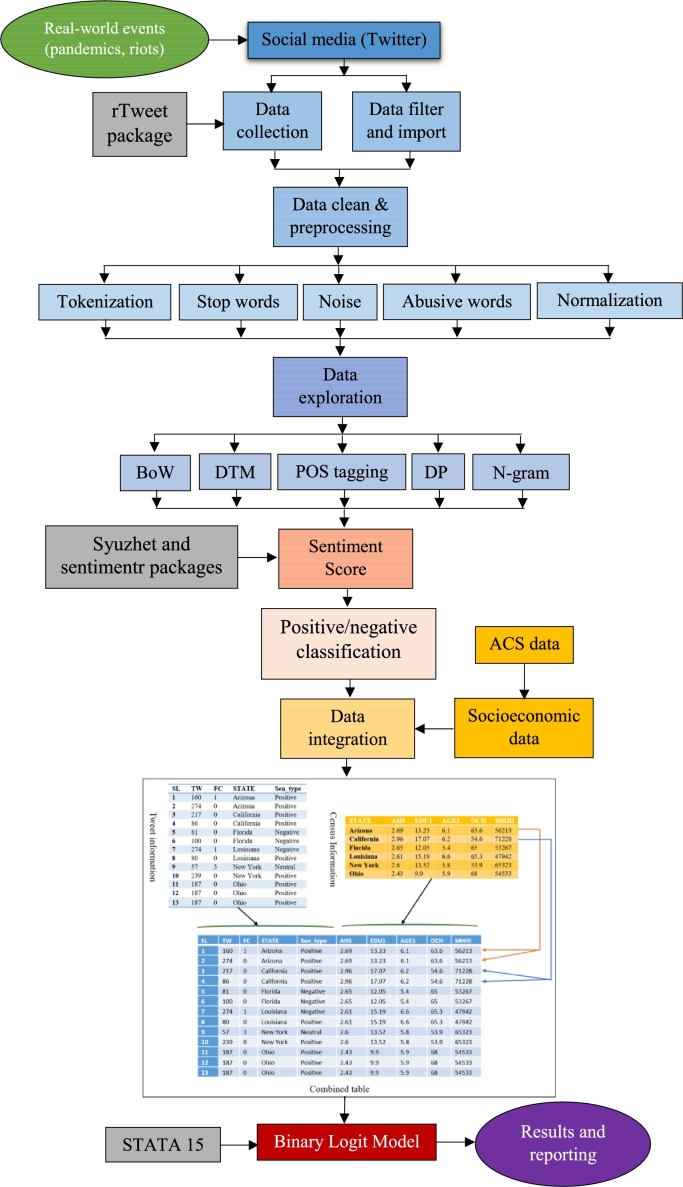
Study design flowchart.

The detailed and systematic methodologies adopted in this study, starting from data collection to model development, results discussion and reporting, as shown in Fig. 3, has been illustrated in Section 3. We collected data from Twitter to understand feelings of the people by using the rTweet package in R and associated Twitter API. However, this method is generally applicable for collecting data from any social media platforms (e.g., Facebook, LinkedIn, Instagram, and news agency) regarding any real-world social events (e.g., man-made and natural disasters, political affairs, religious and racial conflicts). After filtering the tweet's information, the data is saved in CSV format for subsequent prepossessing. Data preprocessing (e.g., cleaning, removing noises) is an important step in text analysis and classification. In the data preprocessing steps, unnecessary words, such as stop words (i.e., pronouns, articles, prepositions such as ‘the’, ‘a’, ‘about’, ‘we’, ‘our’, etc. that do not have any significant contribution to text classification), noises (i.e., punctuation, special characters), and abusive words (i.e., slang words) were removed from the text to improve the efficiency of the system [55], [56].

Moreover, tokenization and normalization techniques are applied to process the text. Tokenization is the process of dividing texts into words, phrases, and symbols which are known as tokens [57]. The main goal of the tokenization is to find out the words or group of words in a sentence, which is the foundation of text analysis. Tokenization is very important for text analysis because a meaningful computation primarily depends on the components (tokens) of the text, not on the full text. For example, if there is a sentence like ‘we have collected data from twitter’. After tokenization, the set of tokens will be: {‘we’, ‘have’, ‘collected’, ‘data’, ‘from’, ‘twitter’}. Normalization transforms words into a common form that allows the computer to identify the same words with similar meaning and remove one of them [56], consequently significantly reduces data size and increases computational efficiency. Lowercasing, stemming and lemmatization are three important and useful methods of text normalization. Lowercasing converts every letter to lowercase and make similar words consistent to each other [55]. For example, lowercasing converts each letter of ‘An’ and ‘an’ to lowercase and helps the computer to identify the two words are identical. Stemming converts words to their base form (stem) [58]. The same word appears in different forms in the texts that are consolidated into the same features [59]. For example, the semantic meaning of ‘read’ and ‘reading’ is the same, thus they are combined as ‘read’ to avoid any confusion. Lemmatization is the most advanced method of text normalization, which replaces words with their same morphological root using a dictionary [56]. Lemmatization is very similar to stemming. However, the basic difference between them is that lemmatization does not produce a stem but replaces the suffix of the word to normalize it with usually a different word suffix [60]. Normalized words with both stemming and lemmatization could be the same. For example, normalized words of ‘reads’, ‘reads’, and ‘reading’ is the same in both methods. However, normalization of the same words could be different in stemming and lemmatization. For instance, the normalized word of computes, computing, computed is ‘comput’ in stemming and compute in lemmatization. After processing the data, different data exploration techniques were used to extract insights from the Twitter data as stated below:

1. **Bag-of-words (BoW)** count different word frequencies in the text to determine the focal point of the text analysis, ignoring the order of the words [55]. For example, the sentence “Although the order of the words is ignored, multiplicity is counted and used to determine the focal point of the text analysis” can be tokenized as {‘Although’, ‘the’, ‘order’, ‘of’, ‘the’, ‘words’, ‘is’, ‘ignored’, ‘multiplicity’, ‘is’, ‘counted’, ‘and’, ‘used’, ‘to’, ‘determine’, ‘the’, ‘focal’, ‘point’, ‘of’, ‘the’, ‘text’, ‘analysis’}. Thus, the corresponding BoW is {1, 4, 1, 2, 1, 2, 1, 1, 1, 1, 1, 1, 1, 1, 1, 1, 1}.
2. **Document term matrix (DTM)** is used to represent text corpus (i.e., collection of texts) in a bag-of-words [56]. DTM is a matrix where rows contain documents, columns contain terms, and cells contain the number of each term that occurred in each text document. TDM allows the researchers to analyze data with vector and matrix algebra, which effectively convert text to numbers. Moreover, text data stored in the DTM format improve memory efficiency and optimize the operation of the data analysis.
3. **Parts-of-speech (POS)** tagging is a basic part of the syntactic analysis, which has numerous applications in NLP [61]. In POS tagging, worlds in the text such as nouns, verbs, articles, and adjectives are identified to understand the context of the text [56]. For example, tagging nouns and proper names researchers identify similar events in news items. It is also a good approach to remove articles and pronouns from the text, which has no meaningful role in the text analysis.
4. **Dependency parsing (DP)** illustrates the syntactic relationship between different tokens [62]. For example: ‘John is an assistant professor at UNCC and he is very popular among the students’. In the example, it is understood that ‘John’ is a nominal subject and ‘professor’ is an adjective, which indicates that John is a professor who teaches students.
5. **The N-gram** technique is used to understand the association between the words. N-gram is not a representation of text, rather presents a set of n-words in the text with their order [55]. N-gram techniques tokenize texts into single words (unigrams), sequences of two words (bigrams), three words (trigrams), and so on to maintain the order of words and syntactical properties [56].

The findings of the exploratory analysis using steps and processes described above helped to gain a clearer understanding of public perspectives on reopening [8]. After data exploration, sentiment score was generated for each tweet by using the R package sentimentr, and sentiments were classified into positive, negative, and neutral based on matching keywords, word sequences, and prewritten lexicons. Twitter data was then integrated with state-wide averaged socioeconomic data collected from the Census to conduct the analysis. A binary logit model was used to evaluate the factors that influence people's sentiment towards a new normal reopening. To perform the analysis, the categorical variable of tweet sentiments was converted to dummy variables where positive sentiment was assigned a value of ‘1’ and negative and neutral sentiments were assigned a value of ‘0’. The category of the tweets was used as the dependent variable in the binary model. On the other hand, different socioeconomic and demographic variables, regional dummies, and Coronavirus cases and deaths have been used as the independent variables in the binary logit model. Finally, the model results are reported and discussed to obtain insights about public reopening sentiment. The following subsection discusses the theoretical framework of the binary logit model.

### 3.3 Binary logit model

In the linear regression model, the response variable Y is quantitative. However, in many situations, the response variable is rather qualitative or categorical, for instance, the sentiment of a tweet could be categorized into positive or negative. The logistic regression model also known as *logit model* classifies the sentiment based on the probability. Assume *X* is the set of features, $\left[x_{1},x_{2},\cdots ,x_{n}\right]$, where *n* is the total number of features in reopening sentiment analysis. pr(Y=1|x) denotes the probability of positive sentiment about reopening given the feature *x*. Conversely, pr(Y=0|x) denotes the probability of negative reopening sentiment given the feature *x*. Using the linear regression model, pr(Y=1|X)=P(X) can be computed as Eq. (1).

(1) $$P(X)=\beta_{0}+\beta_{1}X$$

where $\beta_{0}$ is the intercept and $\beta_{1}$ is the coefficient of *X*. However, Eq. (1) may predict P(X)<0 for *X* close to 0 and P(X)>1 for large value of *X*. To bound P(X) in the range 0 and 1, we use the logistic function as depicted in Eq. (2) [63].

(2) $$P(X)=\frac{e^{\beta_{0}+\beta_{1}X}}{1+e^{\beta_{0}+\beta_{1}X}}$$

Considering the boundary value 0.5, we get the estimated probability ($\overset{ˆ}{P}(X)$) from Eq. (2) using Eq. (3).

(3) $$\overset{ˆ}{P}(X)=\left\{ \begin{array}{cc} 1 & \text{if }P(Y=1|X)\geq 0.5\\ 0 & \text{otherwise} \end{array}\right.$$

After some mathematical manipulation from Eq. (2), we can get Eq. (4).

(4) $$\frac{P(X)}{1-P(X)}=e^{\beta_{0}+\beta_{1}X}$$

After taking log on both side of Eq. (4) we get Eq. (5).

(5) $$\log\left(\frac{P(X)}{1-P(X)}\right)=\beta_{0}+\beta_{1}X$$

The left-hand side of Eq. (5) is called log-odds or logit, which shows that logistic regression model has a logit which is linear in *X*. The coefficients $\beta_{0}$ and $\beta_{1}$ are estimated using the maximum likelihood method. The idea is to estimate the value of $\beta_{0}$ and $\beta_{1}$ for each feature ($x_{i}$) so that it minimizes the difference between the predicted probability $\overset{ˆ}{P}(x_{i})$ and observed probability $P(x_{i})$. The likelihood function can be expressed as Eq. (6) [63].

(6) $$L(\beta_{0},\beta_{1})=\max\left(\prod_{i:y_{i}=1}P(x_{i}).\prod_{i^{\prime }:y_{i^{\prime }}=0}\left(1-P(x_{i^{\prime }})\right)\right)$$

To increase the computation speed, we take log on Eq. (6) and get the log likelihood function as presented in Eq. (7).

(7) $$LL(\beta_{0},\beta_{1})=\max\left(\log\sum_{i:y_{i}=1}P(x_{i})+\log\sum_{i^{\prime }:y_{i^{\prime }}=0}\left(1-P(x_{i^{\prime }})\right)\right)$$

In other words, estimate the values of $\beta_{0}$ and $\beta_{1}$ so that Eq. (7) yields the maximum value.

## 4 Results

The binary logit model is calibrated and estimated using STATA 15 software [64]. The log-likelihood method is used to calculate the coefficients. The final results of the model indicating the impacts of independent variables on the dependent variables are presented in Table 2. The table also reports the standard error, z-value, and probability level (P-value) of the estimates. Many of the coefficients are statistically significant at 0.001, 0.05, and 0.10 levels. However, some of the coefficients with a P-value greater than 0.10 are retained in the model to portray the relationships between dependent and some statistically insignificant independent variables, yet important factors that can influence sentiment of the persons.

**Table 2:** Results of the binary logit model.

Sentiment	Coef.	Std. Err.	z	P>z
TW	0.003	0.001	5.990	0.000
NE	0.253	0.246	1.030	0.303
MW	0.250	0.221	1.130	0.257
WEST	0.702	0.282	2.480	0.013
L_FHH	2.414	2.013	1.200	0.230
AFS	-3.331	1.021	-3.260	0.001
EDU2	0.034	0.026	1.310	0.189
EDU3	0.062	0.054	1.150	0.249
AGE2	0.006	0.099	0.060	0.953
WP	-0.006	0.008	-0.820	0.415
OCH	0.011	0.022	0.520	0.600
PWHI	0.057	0.024	2.360	0.018
LF	0.157	0.058	2.700	0.007
L_POPDEN	0.122	0.098	1.240	0.213
CASES	-0.000003	0.000	-0.180	0.860
PR	0.206	0.081	2.530	0.011
MHHI	-0.00002	0.000	-2.130	0.033
GR	0.003	0.001	2.230	0.026
Constant	-18.042	10.131	-1.780	0.075
LR chi-squared (18)	63.390			
Prob >chi-squared	0.000			
Pseudo R2	0.018			
Log-likelihood	-1700.284			

### 4.1 Characteristics of the tweets

Results presented in Table 2 show that longer tweets (0.003) have significant association with positive sentiment about the US reopening. Thus, people are more likely to post a longer statement on Twitter to express their positive feelings about the reopening the economy with useful information. A study analyzed Twitter data to understand sentiments of the citizens for allocating resources during Hurricane Irma in 2017 in Florida [43]. Upon analyzing data the study found that longer tweets are more likely to have useful information with sentiment contents. It also revealed that popular twitters were more likely to have positive sentiments and less likely to have useful information about the disaster. Thus, longer tweets can provide insightful information on the public sentiments and can be used for crises management during any man-made and natural pandemics.

### 4.2 Regional, family and education association

People live in the Northeast (0.253) and Midwest (0.250) regions are more likely to express positive sentiment about the reopening. However, the relationship is not statistically significant at P-value of 0.05 which indicates that tweets generated from the Northern and Midwest regions have less sentiment contents. Rather, the states located in these two regions, particularly, New York, New Jersey, Illinois, Massachusetts, Pennsylvania, and Michigan are more concerned about the health condition of family members and relatives, and the COVID-19 pandemic due to the higher number of Coronavirus cases and deaths. People live in the West region (0.702) are significantly associated with the positive sentiment towards reopening the economy. The people in the western regions mainly live in California, Nevada, Oregon, and Washington are more interested to reopen the economy because of higher monthly house rent, a higher number of foreign-born people, and low population density than other regions.

Family households (2.414) are more likely to express positive sentiment about the reopening compared to non-family households, though the association is marginally significant. Most of the family households want reopening of the workplaces to earn family expenses. However, they are also concerned about the COVID-19 related health risks associated with the reopening of the workplaces. People with low education levels, high school graduates, and some college (0.034) and associate degree (0.062), are partnered with positive sentiment towards reopening the economy. People with lower levels of education are the worst victim of COVID-19. Most of them have lost their jobs due to the closure of workplaces. Moreover, they have limited options to work from home because most of these low paid jobs are on-site in nature.

### 4.3 Age and income

Young people (age under 18 years) (0.006) are associated with positive sentiment towards reopening the economy. Thus, younger generations are more likely to anticipate a new normal reopening where they can enjoy a COVID-19-restriction-free life. However, the relationship is not statistically significant at P-value of 0.05 which indicates that the effect of younger generation on reopening positive sentiment is not significantly different from zero. Thus, tweets posted by younger people are less likely to have any sentiment contents related to reopening. Previous study recommended to take into consideration of over-reporting tendency of the young generation to gauge the real sentiment of the pandemic [43]. Persons age 16 years and above involved in the labor force (0.157) are significantly associated with positive sentiment of reopening the economy. Similarly, persons under 65 years without health insurance (0.057) are significantly associated with positive sentiment of reopening the economy. Thus, working-age people usually age under 65 years are positive about the reopening despite not having health insurance and tweets posted by them often contain positive facts and figures in favor of a pre-COVID-19 scenario. In contrast, elderly persons having fragile health conditions are more prone to the risk of severe illness from COVID-19 than other age cohorts [13].

Low-income people (0.206) are significantly associated with positive sentiment of reopening the economy. Thus, people with low household income are more interested in reopening the economy due to workplaces closing related job loss [65]. To pay the household bills and meet other household demands they want to go back to work. Similarly, people with high median gross household rent (0.003) are significantly associated with positive sentiment concerning reopening the economy. Thus, people with low-income and high household rent are more prompted and on the support of reopening the economy. In contrast, people with high median household income (-0.00002) are significantly associated with the negative sentiment of reopening the economy. The people of this stratum mostly work at the Information Technology (IT) sector and corporate organizations. Usually, this category of the people dominate higher paid jobs and enjoy work from home facilities. Thus, they are less affected by the COVID-19 compared to the low-income people and consequently less interested to reopen the economy. The results of this study comply with the previous study [43] where they reported a negative association between per capita income and sentiment about a pandemic.

### 4.4 Family size and other factors

Average family size (-3.330) is significantly associated with the negative sentiment of reopening the economy which indicates that families with a large number of members are less likely to reopen the economy. There is a high possibility of COVID-19 infection among the households with larger household members compared to a signal age group (i.e., single household) [66], thus they are less interested to reopen the economy considering the rapid transmission of disease through social contact. Similarly, white people (-0.006) are negatively associated with the reopening sentiment. However, the relationship is marginally significant at P-value of 0.05 which indicates that tweets generated by them are less likely to have any sentiment contents. Similarly, the number of Coronavirus cases (-0.000003) is negatively associated with the reopening sentiment. Thus, with an increasing number of cases, people are less likely to desire reopening the economy. Moreover, the tweets generated from the states with a higher number of Coronavirus cases and deaths have less number of reopening contents. Rather, they are more concerned about the risks associated with COVID-19 and tweets about the severity of the pandemic. People living in household-owned residential units (0.011) and living in the areas with high population density (0.122) are positively associated with reopening the economy, though the relationship is not statistically significant. However, tweets posted by the people living in residential housing units and densely populated areas are less likely to have positive information about reopening the economy. Furthermore, higher population density increases the probability of Coronavirus outbreak due to community transmission by increasing contact rate [67], [68], [69]. Thus, based on tweets generated from the areas with a higher population density, people are less likely to favor reopening the economy, rather they are more apprehended about the severity of the pandemic.

### 4.5 Goodness-of-fit statistics

The overall fit of the calibrated model is evaluated based on several key goodness-of-fit statistics. The likelihood ratio chi-square (LR chi-squared) statistic of the estimated model is 63.39 (Table 2). A lower value of the Chi-square indicates a better fit of the model. P-value (<0.00) of the Chi-square statistic indicates that overall the model is statistically significant and better than a model with no predictors. Thus, the model can significantly fit the observed data because the P-value is less than 0.00.

Other fit statistics also confirm the overall fit of the estimated model (Table 3a). We calculated Pearson chi-square fit statistics to evaluate the overall fit of the model. This is the formal test of the null hypothesis to assess whether the fitted model is correct. The P-value of this hypothesis testing ranges between 0 and 1. P-value specified *α* level (i.e., 0.05) indicates that the model is not statistically significant and acceptable. A higher P-value indicates a better fit of the model. Pearson chi-square test statistics (Prob >chi-squared = 0.20) presented in Table 3a indicates that we cannot reject the null hypothesis and hence, the model is overall fit. In a nutshell, the above discussed fit statistics indicate that the model can adequately fit the observed data.

**Table 3:** Fit statistics and classification summary.

**Table:** Goodness-of-fit test Fit statistics Number of observations 2501 Number of covariate patterns 1968 Pearson chi-squared (1949) 2002.96 Prob >chi-squared 0.20	**Table:** Measure Outcome Correctly classified 56.18% Sensitivity 49.59% Specificity 62.32%
(a) Pearson chi-square fit statistics.	(b) Classification summary.

We also reported classification statistics to evaluate the accuracy and efficiency of the model (Table 3b). Overall, the model can correctly classify 56.18% of the sentiments with 62.32% of specificity (i.e., correctly classify positive sentiment) and 49.59% sensitivity (i.e., correctly classify negative sentiment). Thus, the classification statistics indicate a good prediction accuracy of the model.

**Figure 4:**
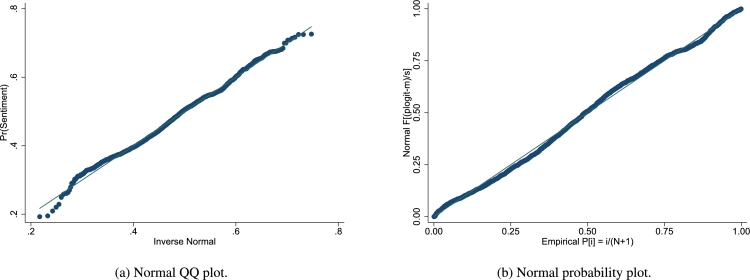
Normal distribution of the residuals.

Normal distribution of the residuals is shown in Fig. 4. The Normal QQ plot and normal probability plot show that the residuals fall perfectly along a linear line at 45^0^ angle. Thus, the residuals are normally distributed. Normal distribution of the residuals indicates that the amount of error in the model is consistent across the observed dataset. Therefore, the predictive capability of the explanatory variables is same for the full range of dependent variables. Moreover, the model can explain all the variations in the dataset sufficiently.

### 4.6 Marginal effects

The results of the binary logit model presented in Table 2 provide an indication of the effects (i.e., positive or negative) of independent variables on the dependent variable. However, it is difficult to interpret the magnitude of marginal effects. Hence, we calculated the marginal effects of explanatory variables using the ‘margins’ command in Stata. Margins make result interpretation easier and report elasticities (i.e., percentage change in the likelihood of positive sentiment for a unit change in an explanatory variable). The results of the marginal effects of explanatory variables have been presented in Table 4. The results in Table 4 indicate that tweet width has a positive impact on reopening the economy. A 1% increase in the tweet width significantly increases positive sentiment by 0.001%. People live in the western regions are significantly associated with reopening sentiment. A 1% increase in tweet generation from the west region of the US increases the probability of positive sentiment for the reopening by 0.175%. The results also indicate that employment status has positive impacts on the reopening. A 1% increase in persons under 65 years without health insurance and persons age 16 years and above involved in the labor force increases reopening positive sentiment by 0.014 and 0.039 unit, respectively. People with low household income are associated with the positive reopening sentiment. A 1% increase in low-income people increases the probability of positive sentiment for reopening by 0.051%. Similarly, high house rent motivates people toward the positive sentiment of reopening the US economy. The result indicates that a 1% increase in house rent leads to the probability of a 0.001% increase in positive sentiment. A 1% change in the family household and population density increases reopening sentiment by 0.603 and 0.030 units, respectively. Furthermore, a 1% increase in people with high school graduates and some college and associate degree increases reopening sentiment by 0.008 and 0.016 units, respectively. In contrast, a 1% increase in average family size significantly reduces the probability of positive sentiment for reopening by 0.832%. Similarly, a 1% increase in household median income reduces reopening positive sentiment by only 0.00001%. However, as evidenced by the magnitude, household income has limited impacts on the opposition of reopening the economy. Interestingly, increasing Coronavirus cases have a limited affect on reopening sentiment - a 1% increase in Coronavirus cases reduces positive sentiment by only 0.000001%. Moreover, a 1% increase in white people reduces reopening positive sentiment by 0.002 units.

**Table 4:** Marginal effect of the explanatory variables.

Sentiment	dy/dx	Std. Err.	z	P>z
TW	0.001	0.0001	5.990	0.000
NE	0.063	0.061	1.030	0.303
MW	0.062	0.055	1.130	0.257
WEST	0.175	0.071	2.480	0.013
L_FHH	0.603	0.503	1.200	0.230
AFS	-0.832	0.255	-3.260	0.001
EDU2	0.008	0.006	1.310	0.189
EDU3	0.016	0.014	1.150	0.249
AGE2	0.001	0.025	0.060	0.953
WP	-0.002	0.002	-0.820	0.415
OCH	0.003	0.005	0.520	0.600
PWHI	0.014	0.006	2.360	0.018
LF	0.039	0.015	2.700	0.007
L_POPDEN	0.030	0.024	1.240	0.213
CASES	-0.000001	0.000	-0.180	0.860
PR	0.051	0.020	2.530	0.011
MHHI	-0.00001	0.000	-2.130	0.033
GR	0.001	0.0003	2.230	0.026

## 5 Discussions and conclusions

COVID-19 pandemic is adversely affecting public and mental health, economy, and mobility of the people. A large number of people are economically and psychologically distressed with job loss and associated fear and uncertainties. Thus, most of the people and businessmen are showing a propensity towards a normal pre-COVID-19 condition. Collecting data from Twitter and Census this study investigated the socioeconomic factor which affect the new normal by virtue of their association with reopening sentiment, by using a binary logit model. Results from the binary logit model explain that tweet width, people living in the western regions of the US, working-class people, gross household rent, and low-income people are positively associated with reopening the economy. On the other hand, average family size and household income are negatively associated with reopening sentiment. However, they have a limited impact. Moreover, the number of Coronavirus cases in a region does not appear to have a significant sentiment association with reopening the economy.

### 5.1 Implications

Several policy recommendations can be drawn from the analysis. By investigating the socioeconomic factor associations for reopening sentiments, this study implicitly pointed out which strata of the people, and which regions bear positive and negative sentiments for reopening the economy. Thus, the federal and state governments and their agencies can use the findings of this study to make better informed decisions, to help secure the economy of the country. The findings can also be used to improve information dissemination, as information categories can impact performance, and increase effectiveness of other preventive measures such as antiseptic and disinfectant protocol (e.g., hand washing, body and nasal spray) are testified to reduce infection of the people [70], [71]. Real-time COVID-19 incidence and socioeconomic characteristics of the people provide essential directions to the policymakers and health professionals to allocate resources for developing vaccines and therapeutics to protect people [72]. This study reported that larger households, which have a greater probability of being composed of a wide range of ages, tend to bear negative sentiment towards reopening the economy. This is most likely in consideration of COVID-19 related health risks for family members in specific age groups, particularly for elderly and those who are very young. However, most of the other socioeconomic factors show support for positive sentiment towards reopening the economy. Opening the economy, especially industries and workplaces are unavoidable to foster economic development of the country. Thus, the decision makers should take adequate protective actions and risk mitigation measures for anticipated health risks and pave the way to a new normal reopening for essential economic activities.

### 5.2 Limitations

Despite timely contribution to the literature, this study has some limitations. First, using twitter data does not represent the complete section of the population. Many people do not use Twitter [73], [74], [75]. Thus, the study using Twitter data may be unable to provide a general idea about the subject matter. Second, sentiment analysis is unable to pick up nuanced or ambiguous meanings (e.g., slang, misspellings, nuanced or ambiguous meanings, Twitter lexicon, inside references, current events, intention, mood) of the tweets which give misleading information [73], [75]. Third, it is very difficult to analyze and identify valuable information from a very large quantity of unstructured and heterogeneous data from social media to acquire useful information for decision making [76]. Fourth, socioeconomic and household information is averaged at the state level which provides a little variation. Thus, a study with fine geographic resolutions (e.g., county, zip code etc.) might provide more insights.

### 5.3 Future research and conclusions

This research uses a novel methodological variation by combining sentiment analytics from Twitter data, with a custom selection of socioeconomic variables from Census data, to create insights that can contribute to developing a clearer understanding of the factors driving post-COVID-19 reopening sentiment. Sentiment and human behavior can be affected by a wide range of factors, including the information propagation formats, and future research could therefore include relevant time-matched news articles and responses to the tweets data for sentiment analysis [17], [77], [78]. This study opens up a valuable stream of research in identifying factors contributing to post-crisis public sentiment using sentiment analysis and can influence future research in policy formation, public mental health, information systems and applications of sentiment analytics. However, though the results of this study are meaningful and align with previous literature, a future study following standard rules of causation analysis would give us more insights about causation effects. A mediation analysis or a structural equation model may help to identify potential causation between dependent and independent variables with possible mediation (i.e., direct and indirect) or moderation effects. Future research could use the current study and build upon it to identify causality and develop causation models.

In summary, this study provides interesting insights to researchers and policymakers, and makes two key contributions: a) the study makes a novel methodological contribution by combining sentiment analytics using Twitter data with Census data, for socioeconomic analysis which can be used for further research, and b) the study provides post-COVID-19 reopening insights into positive sentiment and negative sentiment population segmentations, which can be useful for focused and effective communication and policy management.

## Declarations

### Author contribution statement

M.M. Rahman: Conceived and designed the experiments; Performed the experiments; Analyzed and interpreted the data; Wrote the paper.

G.G.M.N. Ali, J. Samuel: Conceived and designed the experiments; Analyzed and interpreted the data; Wrote the paper.

X.J. Li, K.C. Paul, P.H.J. Chong, M. Yakubov: Analyzed and interpreted the data; Contributed reagents, materials, analysis tools or data; Wrote the paper.

### Funding statement

This research did not receive any specific grant from funding agencies in the public, commercial, or not-for-profit sectors.

### Data availability statement

Data will be made available on request.

### Declaration of interests statement

The authors declare no conflict of interest.

### Additional information

No additional information is available for this paper.
